# Sudden Collapse of Vacuoles in *Saintpaulia* sp. Palisade Cells Induced by a Rapid Temperature Decrease

**DOI:** 10.1371/journal.pone.0057259

**Published:** 2013-02-25

**Authors:** Noriaki Kadohama, Tatsuaki Goh, Miwa Ohnishi, Hidehiro Fukaki, Tetsuro Mimura, Yoshihiro Suzuki

**Affiliations:** 1 Department of Biology, Graduate School of Science, Kobe University, Kobe, Hyogo, Japan; 2 Department of Biological Science, Graduate School of Science, Kanagawa University, Hiratsuka, Kanagawa, Japan; 3 Core Research for Evolutional Science and Technology (CREST), Japan Science and Technology Agency (JST), Tokyo, Japan; Iwate University, Japan

## Abstract

It is well known that saintpaulia leaf is damaged by the rapid temperature decrease when cold water is irrigated onto the leaf surface. We investigated this temperature sensitivity and the mechanisms of leaf damage in saintpaulia (*Saintpaulia* sp. cv. ‘Iceberg’) and other Gesneriaceae plants. Saintpaulia leaves were damaged and discolored when subjected to a rapid decrease in temperature, but not when the temperature was decreased gradually. Sensitivity to rapid temperature decrease increased within 10 to 20 min during pre-incubation at higher temperature. Injury was restricted to the palisade mesophyll cells, where there was an obvious change in the color of the chloroplasts. During a rapid temperature decrease, chlorophyll fluorescence monitored by a pulse amplitude modulated fluorometer diminished and did not recover even after rewarming to the initial temperature. Isolated chloroplasts were not directly affected by the rapid temperature decrease. Intracellular pH was monitored with a pH-dependent fluorescent dye. In palisade mesophyll cells damaged by rapid temperature decrease, the cytosolic pH decreased and the vacuolar membrane collapsed soon after a temperature decrease. In isolated chloroplasts, chlorophyll fluorescence declined when the pH of the medium was lowered. These results suggest that a rapid temperature decrease directly or indirectly affects the vacuolar membrane, resulting in a pH change in the cytosol that subsequently affects the chloroplasts in palisade mesophyll cells. We further confirmed that the same physiological damage occurs in other Gesneriaceae plants. These results strongly suggested that the vacuoles of palisade mesophyll cells collapsed during the initial phase of leaf injury.

## Introduction

Temperature is one of the most important factors affecting plant growth and geographical distribution [1]–[3]. Plants, being immobile, acquire temperature tolerance by altering their physiological conditions before exposure to unfavorable temperatures, such as heat in summer or freezing temperatures in winter [4], [5]. The ability to minimize damage and ensure protection of cellular homeostasis against unfavorable temperature is called acclimation.

During acclimation under seasonal temperature changes, plants alter their fatty acid composition to stabilize membrane fluidity [6], [7]. At higher temperatures, plants accumulate heat shock proteins (HSPs) [8], while at lower temperatures, plants sometimes alter the quantity and type of membrane proteins [9], accumulate antifreeze proteins (AFPs) [10], and/or synthesize compatible solutes against osmotic stress [11]. It is well established that various transcription factors are involved in changes in the expression levels of genes associated with temperature-dependent acclimation [12]–[14].

In addition to seasonal temperature changes, plants are often exposed to sudden changes in environmental temperature. Leaf temperatures change within seconds to minutes in response to changes in sunlight, shade, wind, rainfall, and air temperature [15], [16]. When plants are exposed to drastic changes in temperature, they often become damaged, irrespective of acclimation. This damage is referred to as temperature injury, and can be mediated by effects on membrane fluidity, causing lethal damage to cells [17]–[19]. Likewise, the growth of extracellular ice crystals under freezing conditions results in cell injury [20], [21].

*Saintpaulia* sp. (saintpaulia hereafter), an ornamental species of Gesneriaceae native to Kenya and Tanzania [22], [23], was reported over half a century ago to be damaged by irrigation of cold water onto the leaf surface [24], [25]. It was also reported that as well as saintpaulia, some other Gesneriaceae and Acanthaceae plants are injured by the rapid temperature decrease when cold water is irrigated onto leaves [26]. This leaf tissue damage was considered to be due to chilling injury [27]. However, researchers have shown that the injury is also caused by water at 25°C if the leaf temperature was maintained above 35°C, indicating that temperature difference between the leaf and the water was a more likely cause of the injury [28]. Moreover, the injury was not observed when the leaf temperature was decreased slowly [29]. Thus, the leaf injury of saintpaulia appears to be different from a typical chilling injury.

Leaf cell damage has been mainly investigated in saintpaulia leaves. In injured saintpaulia leaves, palisade mesophyll cells and organelles such as nuclei, mitochondria and chloroplasts were damaged, resulting in cell death [30]. In particular, chloroplasts ruptured and there was a decline in their chlorophyll fluorescence following the temperature decrease [31], [32]. However, the mechanism of this injury is largely unknown, except for a few reports suggesting that reactive oxygen species (ROS) may be involved [33], [34]. The injury is restricted to palisade mesophyll cells; therefore, it is predicted that physiological events resulting in the injury may specifically occur in the palisade mesophyll cells, although the meaning of such a physiological response is unclear. Analysis of this leaf damage could allow us to obtain new insights into the tolerance of plants to rapid drops in temperature.

In the present study, we focus on organelles such as chloroplasts and vacuoles that are thought to be involved in plant cell death [35], [36] to investigate the injury mechanisms in palisade mesophyll cells of saintpaulia. We report that a rapid temperature decrease may affect the vacuolar membrane, resulting in cellular pH changes, followed by organelle damage in the initial phase. We further confirm that the same physiological damage occurs in palisade mesophyll cells of other Gesneriaceae plants, even though these species belong to different phylogenetic groups and have different leaf anatomical structures.

## Materials and Methods

### Plant Material

*Saintpaulia* sp. cv. Iceberg was used as the main material. The 29 species in the 19 associated genera of Gesneriaceae (listed in Table 1) were obtained from the Hyogo Prefectural Flower Center (Hyogo, Japan) and commercial growers. These plants were cultured in damp artificial soil (vermiculite:perlite, 3∶1) at 22°C or 25°C under a 12 h (22°C) or 14 h (25°C) light period at 50 µmol m^−2^ s^−1^ (FL20SS BPN, National, Osaka, Japan). There was no difference between the two different growth conditions for control plants. Fully expanded leaves were detached just before each experiment. In preliminary experiments, we confirmed that leaf detachment did not affect the injury process.

**Table 1:** List of Gesneriaceae species used in this study and GenBank accession numbers for their ribosomal internal transcribed spacer 1 (ITS1) sequences.

Taxa	GenBank accession no.
*Achimenes patens* Benth.	AY182182
*Aeschynanthus gracilis* Parish ex C.B. Clarke	AF349207
*Alsobia dianthiflora* (H.E. Moore et R.G. Wilson) Wiehler	DQ211160
*Alsobia punctata* (Lindl.) Hanst.	DQ211159
*Chirita sinensis* Lindl.	FJ501348
*Chirita tamiana* B. L. Burtt	–
*Chirita* Buch.-Ham. sp. cv. Moon Walker	–
*Codonanthe gracilis* (Mart.) Hanst.	–
*Codonatanthus* W.R.Saylor sp. cv. Vista	–
*Columnea linearis* A. S. Oersted	AF543240
*Columnea* L. sp. cv. Chanticleer	–
*Conandron ramondioides* Sieb. et Zucc. var. *ramondioides*	GU350649
*Episcia lilacina* Hanst.	AY047091
*Gesneria pedicellaris* Alain	AY047049
*Kohleria warszewiczii* (Regel) Hanst.	AY702379
*Kohleria* Regel sp. cv. Bach	–
*Lysionotus pauciflorus* Maximowicz var. *pauciflorus*	AB498566
*Nematanthus gregarius* D.L. Denham	–
*Nematanthus maculatus* (Fritsch) Wiehler	–
*Petrocosmea flaccida* Craib	–
*Primulina tabacum* H. F. Hence	GQ497202
*Saintpaulia* Wendl sp. cv. Iceberg	AF316923^a^
*Seemannia sylvatica* (Kunth) Hanst.	AY702365
*Sinningia speciosa* (Lodd.) Hiern	AY372337
*Sinningia* Nees sp. cv. Tinkerbells	–
*Streptocarpus formosus* (Hilliard et B.L. Burtt) T.J. Edwards	AF316983
*Streptocarpus kentaniensis* Britten et Story	AF316974
*Streptocarpus saxorum* Engler	AF316914
*Streptocarpus* Lindl sp. cv. Popsicle	–

a Saintpaulia cultivars are mainly derived from the original species *Saintpaulia ionantha* [23]. Therefore, we used the ITS 1 sequence of *Saintpaulia ionantha* to estimate the position of cv. “Iceberg” in the phylogenetic trees.

### Estimation of Leaf Injury after a Temperature Decrease

To change leaf temperatures, detached leaves were immersed in temperature-controlled water in a circulation bath (Thermo Minder SM-05, Taitec, Koshigaya, Japan). Each leaf was tightly packed in a plastic bag to prevent the leaves from coming into direct contact with the water. Leaf surface temperature was measured by a thermocouple placed on the surface of the leaf. When a leaf kept in a bath at a particular temperature (T1) for 60 min was transferred to another bath of different temperature (T2) for 1 min, leaf temperature decreased from T1 to T2 within 10 s. When cold water was added gradually to the water bath, both bath and leaf temperatures decreased simultaneously at −5°C min^−1^. After the temperature change treatment, the tested leaf was wrapped with aluminum foil and kept at 22°C for an appropriate period to avoid leaf shrinkage. The leaf was then removed from the aluminum foil and images were taken using a digital camera (COOLPIX4500, Nikon. Tokyo, Japan). The images were analyzed with the PC program LIA for Win32 (http://www.agr.nagoya-u.ac.jp/~shinkan/LIA32/index-e.html). The ratio of damaged leaf area to the total leaf area (referred to as ‘injury severity’) was used to estimate relative injury.

To control pre-incubation temperature and period, packed leaves were incubated in the bath at 30°C or 20°C for 5, 10, 20, 30, 60 min. After incubation, leaves were transferred to another bath maintained at 10°C for 1 min. After the temperature change treatment, the ‘injury severity’ of each tested leaf was measured as mentioned above.

### Preparation and Observation of Leaf Sections

Leaves before and after 24 h of a rapid temperature decrease from 30°C to 10°C were fixed in FAA (formalin:acetic acid:50% ethanol, 5∶5:90) and embedded in a methacrylate resin (Technovit 7100, Heraeus Kulzer GmbH, Wehrheim, Germany). Sections of 5 µm thickness were made using a microtome (Nippon Optical Works, Tokyo, Japan) and stained with 0.1% toluidine blue before being covered with a mounting solution (Entellan neu, Merck KGaA, Darmstadt, Germany). The sections were observed under a light microscope (BX51, Olympus, Tokyo, Japan).

### Chloroplast Isolation

Chloroplasts were isolated with a continuous Percoll gradient centrifugation method as described previously [37]. Fully expanded leaves (10 g) were infiltrated into enzyme solution [2% Cellulase R-10 (Yakult pharmaceutical industry, Tokyo, Japan), 1% Macerozyme R-10 (Yakult pharmaceutical industry), 10 mM Mes, 1 mM CaCl_2_•2 H_2_O, and 0.6 M sorbitol] at 30°C. After 10 min of infiltration, the adaxial side of the leaves (palisade layer and upper epidermis) was separated from the abaxial side (spongy layers and lower epidermis) with tweezers. The separated adaxial surfaces were collected and homogenized at room temperature in 30 ml of a buffer solution [0.35 M sorbitol, 50 mM HEPES-Tris (pH 7.5), 0.1% BSA, 1 mM MgCl_2_, 1 mM MnCl_2_, 2 mM NaNO_3_, 1 mM NaH_2_PO_4_, 2 mM EDTA (2 Na), and 2 mM Na iso-ascorbate] using an homogenizer. The homogenates were filtered through four-layer cheesecloth, and the filtrate was centrifuged at 2,500 ***g*** for 2 min. The pellet was resuspended in the buffer solution and centrifuged again. The washed pellet was resuspended in 1 ml of the buffer solution and mixed with 30 ml of Percoll solution [50% (v/v) Percoll, 5% (w/v) PEG 6000, 1% (w/v) Ficoll 400, and the same ingredients as the buffer solution] in a tube. The tube was then centrifuged at 39,800 ***g*** for 20 min. Chloroplasts were separated as two green layers near the top and the bottom of the tube. The bottom layer containing intact chloroplasts was collected and resuspended in 15 ml of the buffer solution. The suspension was centrifuged at 2,500 ***g*** for 2 min. The pellet was resuspended in 2 ml of the buffer solution. Chloroplast intactness was assessed as 79.1% (n = 3) by the Hill reaction [38].

### Measurement of Chlorophyll Fluorescence during Temperature Decrease

Chlorophyll fluorescence of detached leaves and isolated chloroplasts was determined with a pulse amplitude modulated fluorometer (PAM; PAM-200, Heinz Walz GmbH, Effeltrich, Germany). A leaf sample (10×10 mm) was cut with a razor and the adaxial side was set on the PAM measuring head. A glass chamber, whose temperature was controlled by circulating water, was set on the abaxial side of the leaf piece. To change the temperature, the water temperature circulating within the chamber was controlled. The temperature measured by a thermocouple was quickly adjusted to the water temperature in the chamber. The leaf was kept in the dark at 25°C for 60 min before the measurement. Minimum fluorescence (F_0_ and F_0_’) was determined by measuring light of 3 µmol photons m^−2^ s^−1^ in the dark-adapted leaves and by actinic light (which facilitates photosynthesis) of 80 µmol photons m^−2^ s^−1^ in the light-adapted leaves, respectively. Maximal fluorescence (Fm and Fm’) was determined by a saturating pulse of 0.6 s duration. Changes in the quantum yield of photosystem II (øII) = (Fm’ – F)/Fm’ and in the non-photochemical quenching (NPQ) = (Fm – Fm’)/Fm’ during the temperature change were estimated with the DA-TEACH software (Heinz Walz GmbH). For measurement of chlorophyll fluorescence of isolated chloroplasts, an aliquot of the chloroplast suspension on a well slide glass was set on the PAM measuring head. For comparison, the fluorescence of chloroplasts suspended in a similar buffer solution, but at different pH [0.35 M sorbitol with 50 mM MES-NaOH (pH 5.0 and 6.0) or 50 mM HEPES-KOH (pH 7.6), as well as 1 mM MgCl_2_, 1 mM MnCl_2_, and 2 mM EDTA] was also observed with a microscope. Isolated chloroplasts were resuspended in the buffer solutions and left for 10 min prior to observation.

### Monitoring of Intracellular pH with pH-sensitive Fluorescent Dye

Leaf cross-sections, 100 µm thick, were prepared using a microtome (Plant Microtome MT-3, Nippon Medical and Chemical Instruments, Osaka, Japan) and were stained with 1 µM BCECF-AM (Molecular Probes, Eugene, OR, USA) in a buffer solution (50 mM HEPES-Tris, pH 7.8) for 60 min at 25°C. The sections were placed in a fluorescent dye-free buffer solution on a MATS-555R-IM temperature controller (Tokai Hit, Shizuoka, Japan) on the stage of a confocal laser scanning microscope (FV1000-D, Olympus, Tokyo, Japan). The temperature was kept at 25°C for 10 min and then decreased to 5°C for 1 min followed by rewarming to 25°C. BCECF_AM fluorescence intensities were measured using the ImageJ software (W. Rasband, National Institutes of Health, Bethesda, MD, USA).

### Observation of Vacuolar Dynamics during Cell Injury

To visualize the dynamics of vacuoles during cell injury, vacuolar membranes of palisade mesophyll cells were labeled with the fluorescent membrane probe FM1-43 (Molecular Probes). FM1-43 dye is first taken up by the plasma membrane, and then by the vacuolar membrane via endocytosis [39]. Leaf cross-sections 100 µm thick were prepared with the microtome and then stained with 5 µM FM1-43 in a buffer solution (50 mM HEPES-Tris, pH 7.8) for 60 min at 25°C. To label the vacuolar membrane, the stained sections were incubated in dye-free buffer solution for 180 min at 25°C, after which temperature treatments were applied for 1 min on the stage of the microscope, as described above.

### Phylogenetic Analyses

The phylogenetic relationships of 18 species of Gesneriaceae were examined based on ribosomal DNA internal transcribed spacer 1 (ITS1) sequences. All ITS1 sequence data were obtained from GenBank; accession numbers are listed Table 1. The ITS1 sequences were aligned using the ClustalW option in the analysis software package MEGA5 [40]. A molecular phylogenetic tree was constructed with MEGA5 using the neighbor-joining [41] and maximum likelihood methods [42]. The robustness of the resulting phylogenetic tree was tested by bootstrap analysis with 1000 replicates [43]. The evolutionary distances were computed using the maximum composite likelihood method [44]. Two species of Acanthaceae, which is closely related to Gesneriaceae, *Crossandra infundibuliformis* and *Hypoestes phyllostachya* (GenBank accession nos. AF169842 and AF169754, respectively), were used as an outgroup.

## Results

### Leaf Injury after Rapid Temperature Decrease in Saintpaulia

**Figure 1:**
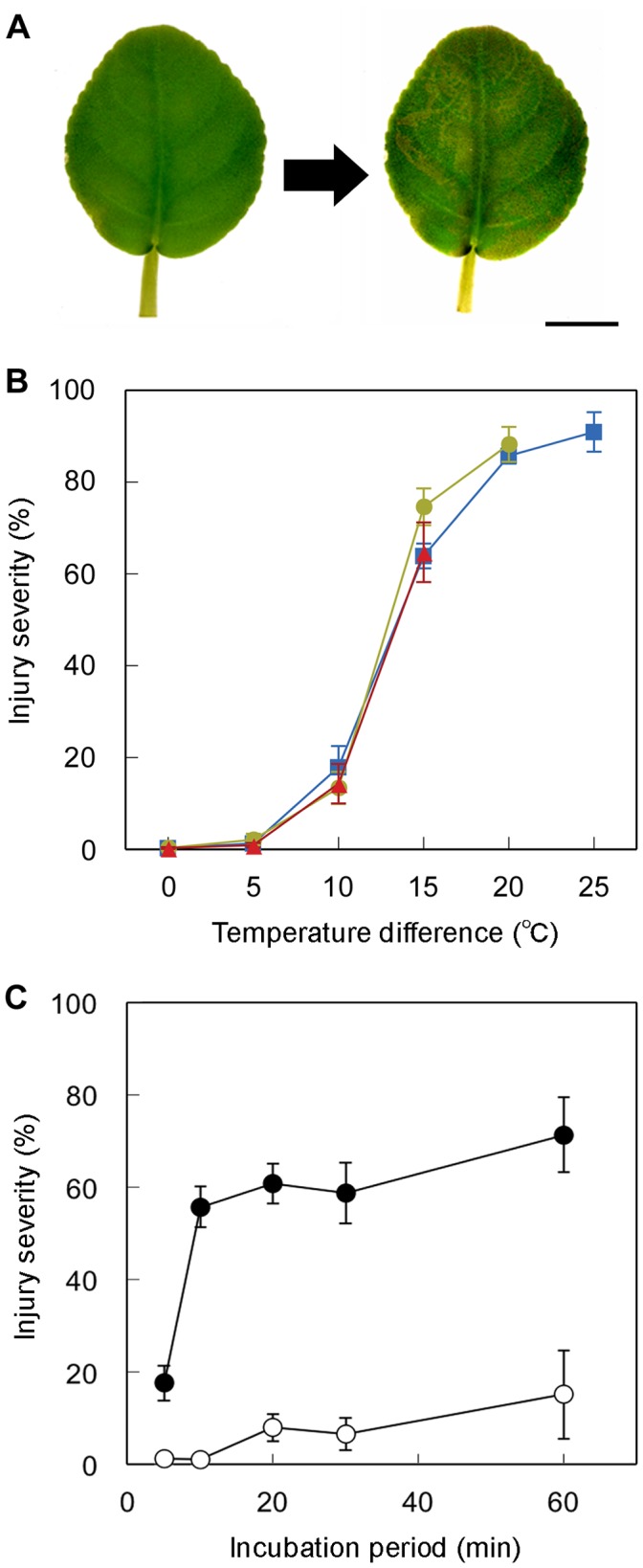
Leaf discoloration and injury severity of saintpaulia. (A) Leaf color change of saintpaulia before (left) and 24 hours after (right) a rapid temperature decrease from 25°C to 5°C. Scale bar = 2 cm. (B) Severity of leaf injury after rapid temperature decrease of leaves initially kept at 30°C (*squares*), 25°C (*circles*), and 20°C (*triangles*). (C) Severity of injury when leaves pre-incubated at 30°C (*closed circles*) or 20°C (*open circles*) for 5, 10, 20, 30, 60 min were rapidly adjusted to 10°C. The points and associated bars in (B) and (C) indicate mean severity and standard error (n = 5).

We first confirmed that a rapid temperature decrease induced leaf injury in saintpaulia in our measuring system. Figure 1A shows the leaf damage resulting from a rapid temperature decrease from 25°C to 5°C. Leaves became brown 24 h after the temperature decrease. The severity of leaf injury after the temperature treatments was estimated as the proportion of damaged area to total leaf area (Fig. 1B). When the leaf temperature rapidly decreased from 30°C to 5, 10, 15, 20 and 25°C, the injury severities were 90.9, 85.7, 63.9, 17.9, and 1.3%, respectively. The severities resulting from lower initial temperatures were also measured. These results agreed with the former observations that the injury severity at a given temperature was affected by the initial leaf temperature.

We attempted to determine the effects of pre-incubation period on leaf injury. Leaves detached from plants growing at 25°C were incubated at 30°C or 20°C for various periods. When leaves incubated at 30°C for 5, 10, 20, 30, 60 min were rapidly cooled to 10°C, the injury severities were 17.6, 55.6, 60.7, 58.6, and 71.3%, respectively (Fig. 1C). The severity of injury to leaves that were incubated at 20°C and cooled to 10°C was mild; incubation of leaves for 60 min resulted in an injury severity of 15.1%. Although the ranges in temperature decrease were the same, the injuries were more severe following pre-incubation at 30°C for over 10 min. These results suggested that the physiological condition of the leaf was drastically changed within 10 min of pre-incubation.

### Changes in Chlorophyll Fluorescence during Injury

**Figure 2:**
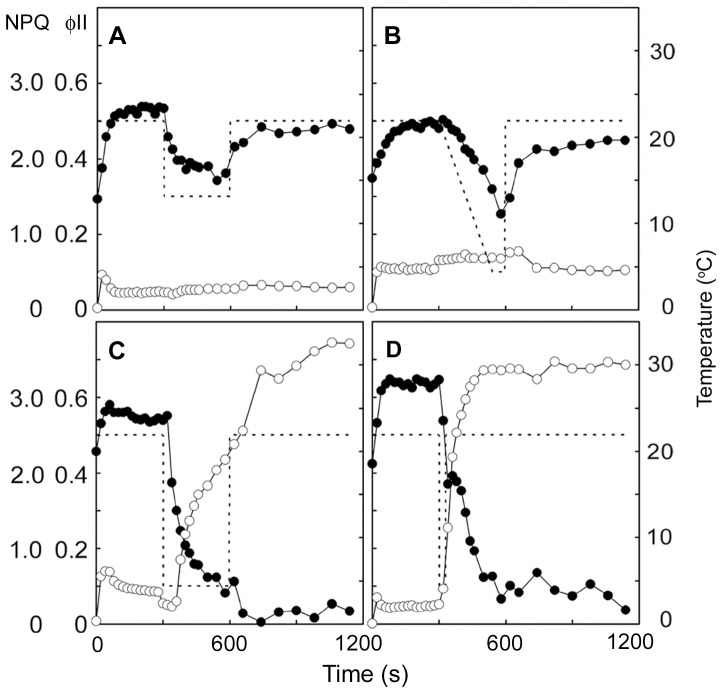
Changes in chlorophyll fluorescence during injury in saintpaulia leaf. Solid lines show quantum yield of PS II (øII; *closed circles*) and non-photochemical quenching (NPQ; *open circles*), while broken lines indicate leaf temperature. These indices were measured during a rapid temperature decrease from 25°C to 15°C followed by rewarming to 25°C (A), during a gradual decrease from 25°C to 5°C (B), during a rapid decrease from 25°C to 5°C and kept at 5°C for 300 s followed by rewarming to 25°C (C), and during a rapid decrease from 25°C to 5°C and kept at 5°C for 30 s followed by rewarming to 25°C (D).

The decline of chlorophyll fluorescence from injured palisade mesophyll cells has been observed with a fluorescence microscope [32]. In the present study, we assessed the decline of chlorophyll fluorescence during injury using two indices of photosynthesis, the quantum yield of PS II (øII) and non-photochemical quenching (NPQ), using a pulse amplitude modulated fluorometer (PAM). When the leaf temperature was maintained at 25°C for 15 min, øII and NPQ did not change (data not shown). Following a rapid temperature decrease from 25°C to 15°C, øII decreased by 28% but increased to the initial value when the leaf temperature returned to 25°C, whereas NPQ did not change (Fig. 2A). When the leaf temperature was decreased slowly from 25°C to 5°C at a rate of 5°C min^−1^, øII decreased by 50% and recovered to ∼90% when the leaf temperature returned to 25°C, whereas NPQ did not change (Fig. 2B). When the temperature was reduced rapidly from 25°C to 5°C and kept at 5°C for 300 s, øII decreased by 85% and continued to decrease after the temperature returned to 25°C. Concurrently, NPQ increased and remained high (Fig. 2C). Similar changes of øII and NPQ were also observed when the temperature was decreased rapidly from 25°C to 5°C, kept constant at 5°C for 30 s, and then returned to 25°C (Fig. 2D).

**Figure 3:**
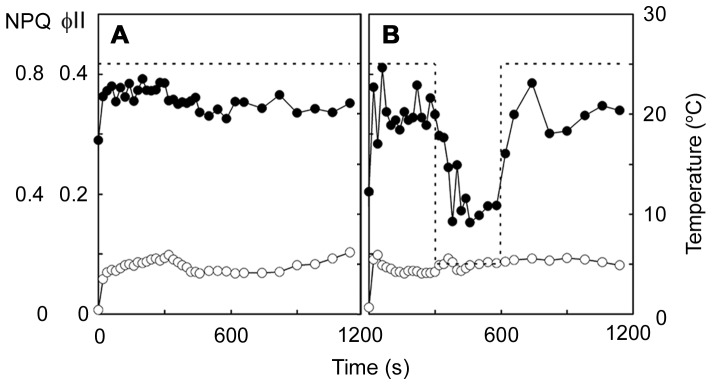
Chlorophyll fluorescence of chloroplasts isolated from palisade mesophyll cells of saintpaulia leaf. Quantum yield of PSII (øII) and non-photochemical quenching (NPQ) of isolated chloroplasts estimated from changes in chlorophyll fluorescence. Solid lines show øII (*closed circles*) and NPQ (*open circles*), while broken lines show temperature. These indices were measured at 25°C (A), and during a rapid decrease from 25°C to 5°C and kept at 5°C for 300 s followed by rewarming to 25°C (B).

To confirm whether chloroplasts were directly affected by the temperature decrease, we isolated chloroplasts from saintpaulia leaves and monitored øII and NPQ. Because the chloroplasts of palisade mesophyll cells were bigger than those of spongy mesophyll cells (data not shown), they could be clearly distinguished under the microscope. The chloroplast suspensions used in this study contained 90.2% palisade mesophyll cell chloroplasts; therefore, we can assume that changes in chlorophyll fluorescence originated in the chloroplasts of these cells. Under the microscope, chlorophyll fluorescence was observed even after 10 min of the temperature decrease (data not shown). øII and NPQ, monitored using PAM, did not change when the temperature was maintained at 25°C for 15 min (Fig. 3A). During the rapid temperature decrease from 25°C to 5°C, øII decreased by 59% but recovered to 93% after rewarming, and NPQ did not change markedly (Fig. 3B). These results indicated that the damage of chloroplasts was not directly induced by the temperature decrease.

### Changes in Intracellular pH Induced by Rapid Temperature Decrease

**Figure 4:**
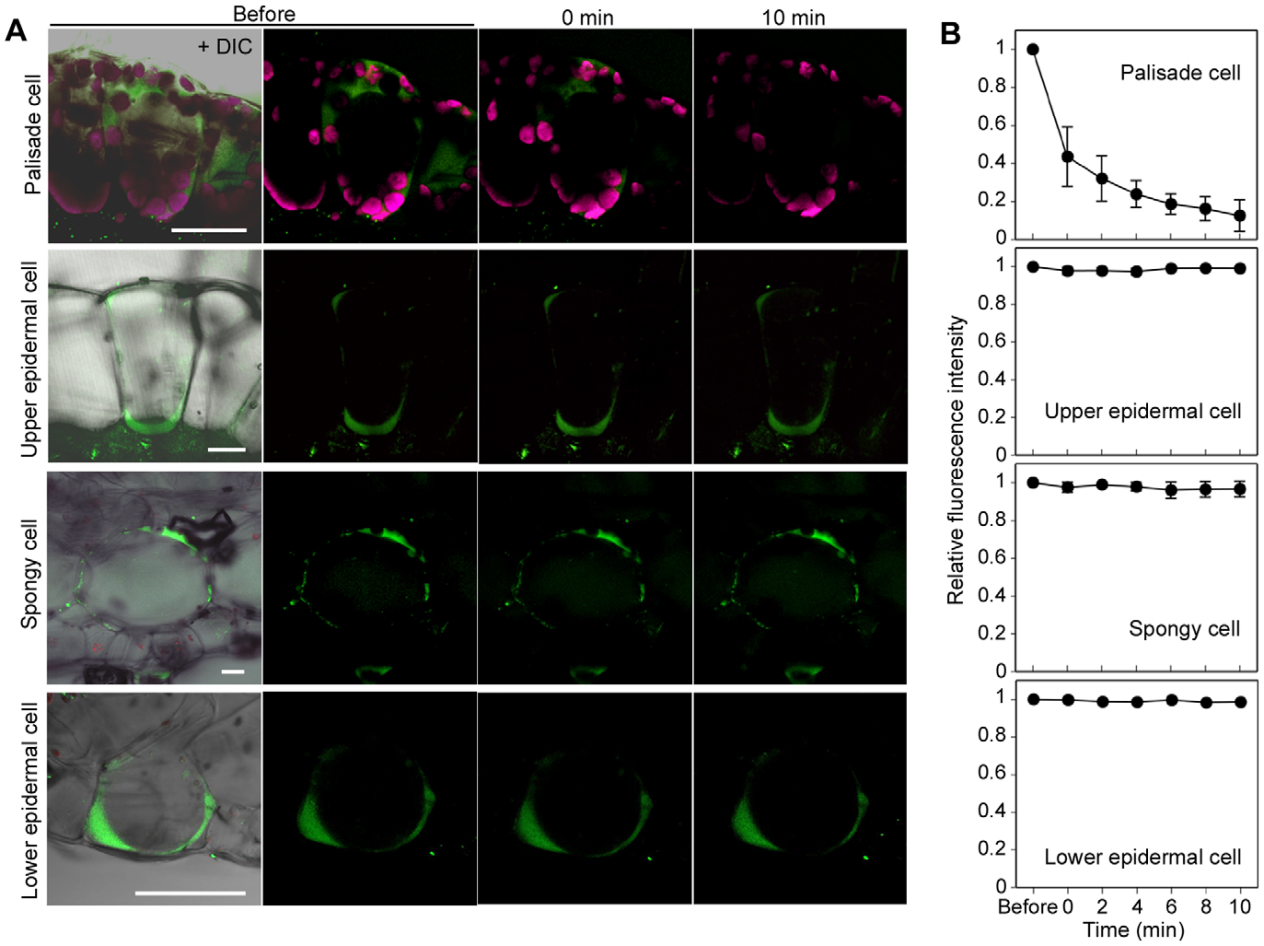
Cytosolic pH changes in palisade mesophyll, spongy mesophyll, and epidermal cells of saintpaulia leaf. (A) Palisade mesophyll, spongy mesophyll, and upper and lower epidermal cells stained with BCECF-AM. BCECF-AM (green) and chlorophyll fluorescence (magenta) were observed before, immediately after (0 min), and 10 min after the temperature was decreased from 25°C to 5°C and kept at 5°C for 1 min. Scale bar = 40 µm. (B) Relative changes of BCECF-AM fluorescence intensity after a temperature decrease. The points and associated bars indicate mean intensity and standard error (n = 3).

Since isolated chloroplasts were not directly damaged by decreasing temperature, we supposed that the intracellular environment of the chloroplasts may be altered by the temperature decrease. Changes in cytoplasmic pH were estimated with a pH-sensitive fluorescent dye, BCECF-AM. The BCECF dye emitted green fluorescence (535 nm) above pH 6.4 when excited at 488 nm. Prior to the rapid temperature decrease from 25°C to 5°C, green fluorescence of BCECF was observed in the cytosol, but was low in the vacuole of saintpaulia palisade mesophyll cells (Fig. 4A). Immediately after the rapid temperature decrease, the fluorescence from the thin layer inside the palisade mesophyll cells became weaker, and virtually disappeared within 10 min. The fluorescence intensity decreased by 87% after 10 min of the temperature decrease (Fig. 4B), whereas the intensity was maintained without the temperature decrease (Fig. S1). In the spongy mesophyll and epidermal cells, the fluorescence of BCECF-AM did not decrease even after the temperature decrease (Fig. 4A, B). These results showed that the pH in the cytosol of palisade mesophyll cells became more acidic after the temperature decrease.

### Effects of Surrounding pH on Isolated Chloroplasts

**Figure 5:**
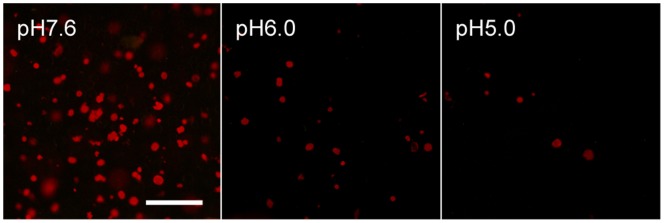
Effects of surrounding pH on isolated chloroplasts. Fluorescent microscopic images of chloroplasts isolated from saintpaulia leaf suspended in buffer solution at pH 7.6 (left), pH 6.0 (middle), and pH 5.2 (right). Scale bar = 50 µm.

A decline of chlorophyll fluorescence was observed concurrently with intracellular pH changes in palisade mesophyll cells (Fig. 4A). To confirm whether chloroplasts were affected by the surrounding solution pH, we observed the chlorophyll fluorescence when isolated chloroplasts were suspended in buffer solutions with varying pH. Under the microscope, the chlorophyll fluorescence of isolated chloroplasts suspended in buffer solution was lower at pH 6.0 than at pH 7.6, and even lower at pH 5.0 (Fig. 5). These results indicated that the decline of chlorophyll fluorescence during the rapid temperature decrease could be induced by acidification of the cytosol.

### Vacuolar Dynamics during Cell Injury

**Figure 6:**
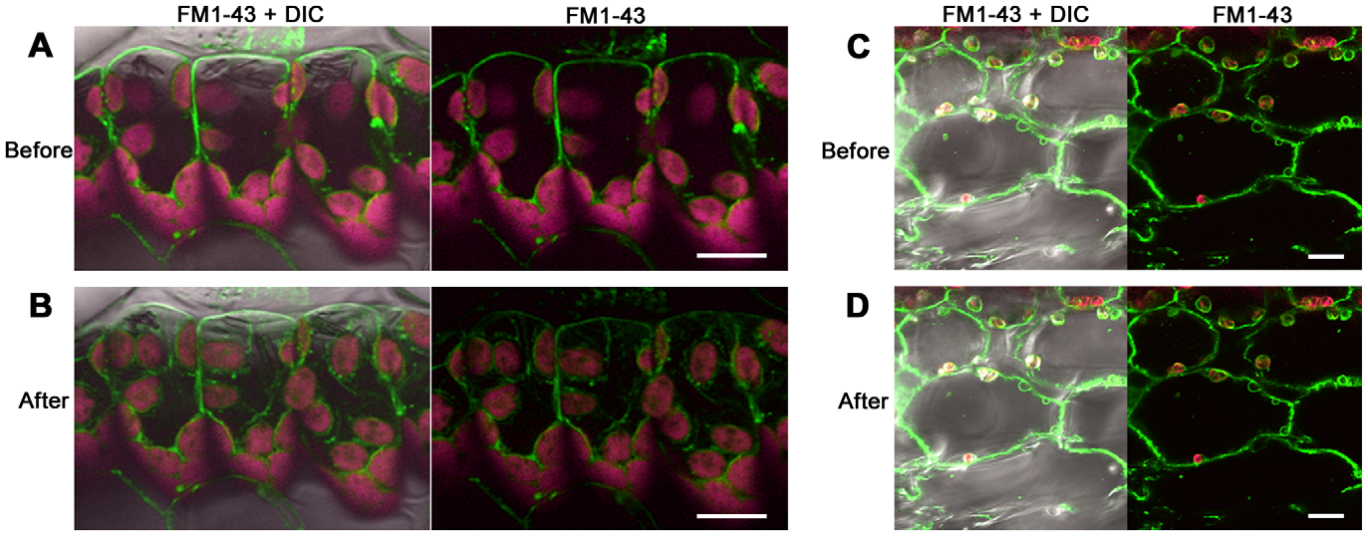
Vacuolar dynamics after a rapid temperature decrease. Vacuolar membrane labeled with FM1-43 (green) and chlorophyll fluorescence (magenta) in palisade mesophyll cells of saintpaulia leaf were observed before (A) and 10 min after (B) a rapid temperature decrease from 25°C to 5°C and kept at 5°C for 1 min. Scale bar = 20 µm. FM1-43 and chlorophyll fluorescence in spongy mesophyll cells observed before (C) and after (D) a rapid temperature decrease from 25°C to 5°C. Scale bar = 40 µm.

Since the cytosolic pH changed during cell injury, we predicted that there may have been leakage of acidic contents from the vacuoles in palisade mesophyll cells. To determine whether the vacuoles of the palisade mesophyll cells were affected by the rapid temperature decrease, we observed the vacuolar dynamics during cell injury using FM1-43. Prior to the rapid temperature decrease from 25°C to 5°C, the FM1-43 fluorescence of the membrane surrounding the large vacuole in palisade mesophyll cells was monitored (Fig. 6A). Chloroplasts emitting autofluorescence were seen at the outer surface of fluorescently labeled membrane. After 10 min of the temperature decrease, FM1-43 fluorescence also appeared inside the vacuole and around the outside of the chloroplasts (Fig. 6B). In spongy mesophyll cells, where cytosolic acidification was not observed (Fig. 4), the fluorescence pattern of FM1-43 remained unchanged even after the rapid temperature decrease (Fig. 6C, D). The vacuolar membrane of palisade mesophyll cells showed dynamic changes during the cell injury, which suggested that the membrane may have collapsed following the rapid temperature decrease.

### Leaves of Other Gesneriaceae Species Injured by Rapid Temperature Decrease

Leaf injury by temperature changes have been reported in other Gesneriaceae and Acanthaceae species [26]. We investigated whether those temperature-sensitive plants have similar physiological mechanisms to saintpaulia.

**Figure 7:**
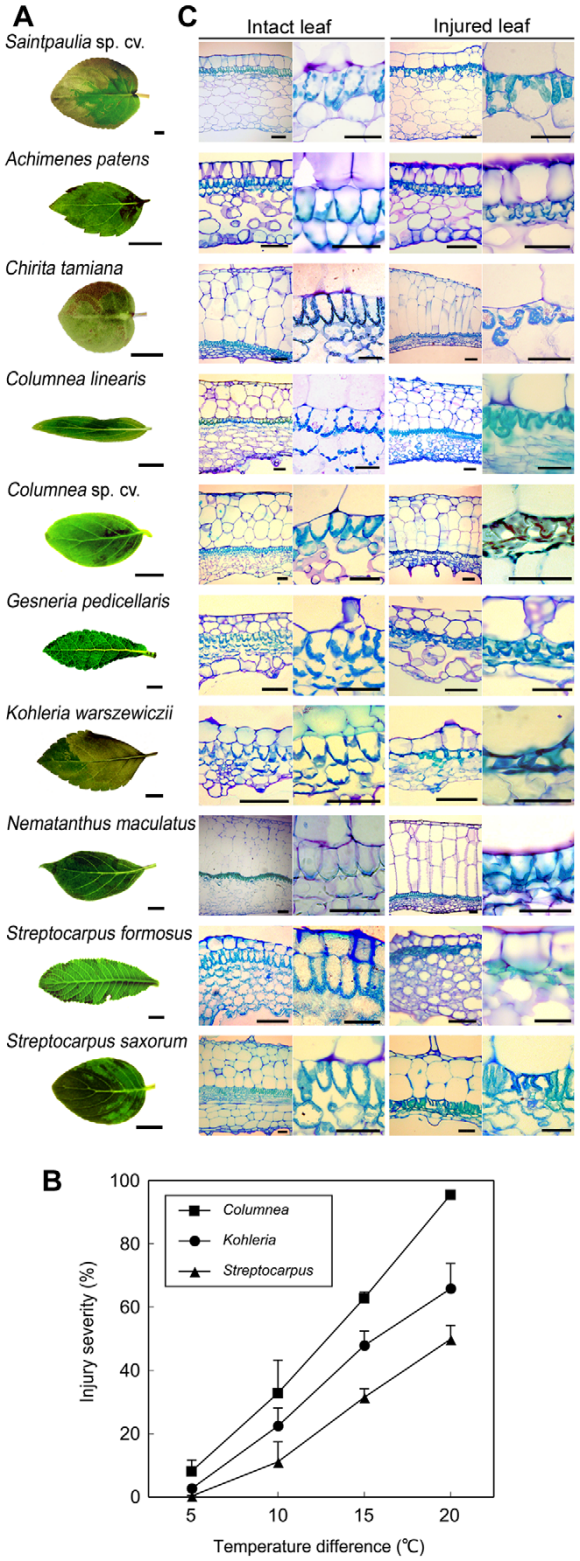
Leaf discoloration and injury severity of Gesneriaceae plants. (A) Discolored leaves of 10 Gesneriaceae plants after a rapid temperature decrease from 30°C to 10°C. (B) Severity of leaf injury in *Columnea* sp. (*squares*), *Kohleria warszewiczii* (*circles*), and *Streptocarpus saxorum* (*triangles*) after a rapid temperature decrease. Leaves initially kept at 30°C to 15°C were rapidly adjusted to 10°C. The points and associated bars indicate mean severity and standard error (n = 3). (C) Cross sections of intact or discolored leaves of 10 Gesneriaceae plants. From left to right: whole leaf sections of intact leaves, magnified view of palisade mesophyll cells of intact leaves, whole leaf sections of discolored leaves, and magnified view of palisade mesophyll cells of discolored leaves. Scale bars = 200 µm in whole-leaf images and 100 µm in magnified palisade mesophyll images.

The leaves of 29 species in the 19 associated genera of Gesneriaceae were tested to investigate their sensitivity to rapid temperature decrease. The leaves of 10 species were sensitive to a rapid temperature decrease from 30°C to 10°C, which resulted in leaf injury (Fig. 7A). The injured areas discolored within 10 min of the treatment. When the leaf temperature of the 10 sensitive species was slowly decreased from 30°C to 10°C at a rate of 5°C min^−1^, the leaves were not injured (data not shown). Although the decrease of temperature was within the same range, a slow decrease did not result in leaf discoloration.

The injury severity after the temperature decrease was estimated in three sensitive species; *Columnea* sp., *Kohleria warszewiczii*, and *Streptocarpus saxorum* (Fig. 7B). When leaf temperature was rapidly decreased from 30°C to 10°C, 95.5%, 65.8%, and 49.9% of the total leaf area was discolored in *C.* sp., *K. warszewiczii*, and *S. saxorum* leaves, respectively. When leaf temperatures were decreased from 15°C to 10°C, the injury severities were 8.2%, 2.7%, and 0.4%, respectively. The lower the initial temperature of the leaves, the lower the injury severities were, even though the leaf temperatures were decreased to the same level (10°C). The injury severity at 10°C was affected by the initial leaf temperature before the temperature decrease. This result was in good agreement with the temperature conditions resulting in leaf injury of saintpaulia shown in Figure. 1.

Transverse sections of injured leaves were observed microscopically and compared with those of intact leaves (Fig. 7C). We observed 10 sensitive species; *Achimenes patens*, *Chirita tamiana*, *Columnea linearis*, *Columnea* sp., *Gesneria pedicellaris*, *Kohleria warszewiczii*, *Nematanthus maculatus*, *Saintpaulia* sp., *Streptocarpus formosus*, and *Streptocarpus saxorum*. The leaves of *C*. *tamiana*, *C*. *linearis*, *C.* sp., *N. maculatus*, *G. pedicellaris*, and *S.* s*axorum* had one to five cell layers of hypodermis beneath the upper epidermis. *A. patens*, *K. warszewiczii*, *S.* sp., and *S. formosus* did not have a hypodermis layer. Palisade mesophyll was layered beneath the upper epidermis or hypodermis. Between the palisade mesophyll and lower epidermis, three to six layers of spongy mesophyll were observed. Both palisade mesophyll and spongy mesophyll cells contained chloroplasts. After 24 h following the temperature decrease from 30°C to 10°C, the palisade mesophyll cells were injured and the chloroplasts were disordered, whereas no notable changes were observed in the upper and lower epidermis, hypodermis, and spongy mesophyll. Injury to the palisade mesophyll cells was observed in all discolored leaves, regardless of the variation in leaf anatomical structure. This result indicated that only palisade mesophyll cells were sensitive to the rapid temperature decrease and that the difference in tissue structures was not correlated with temperature susceptibility.

**Figure 8:**
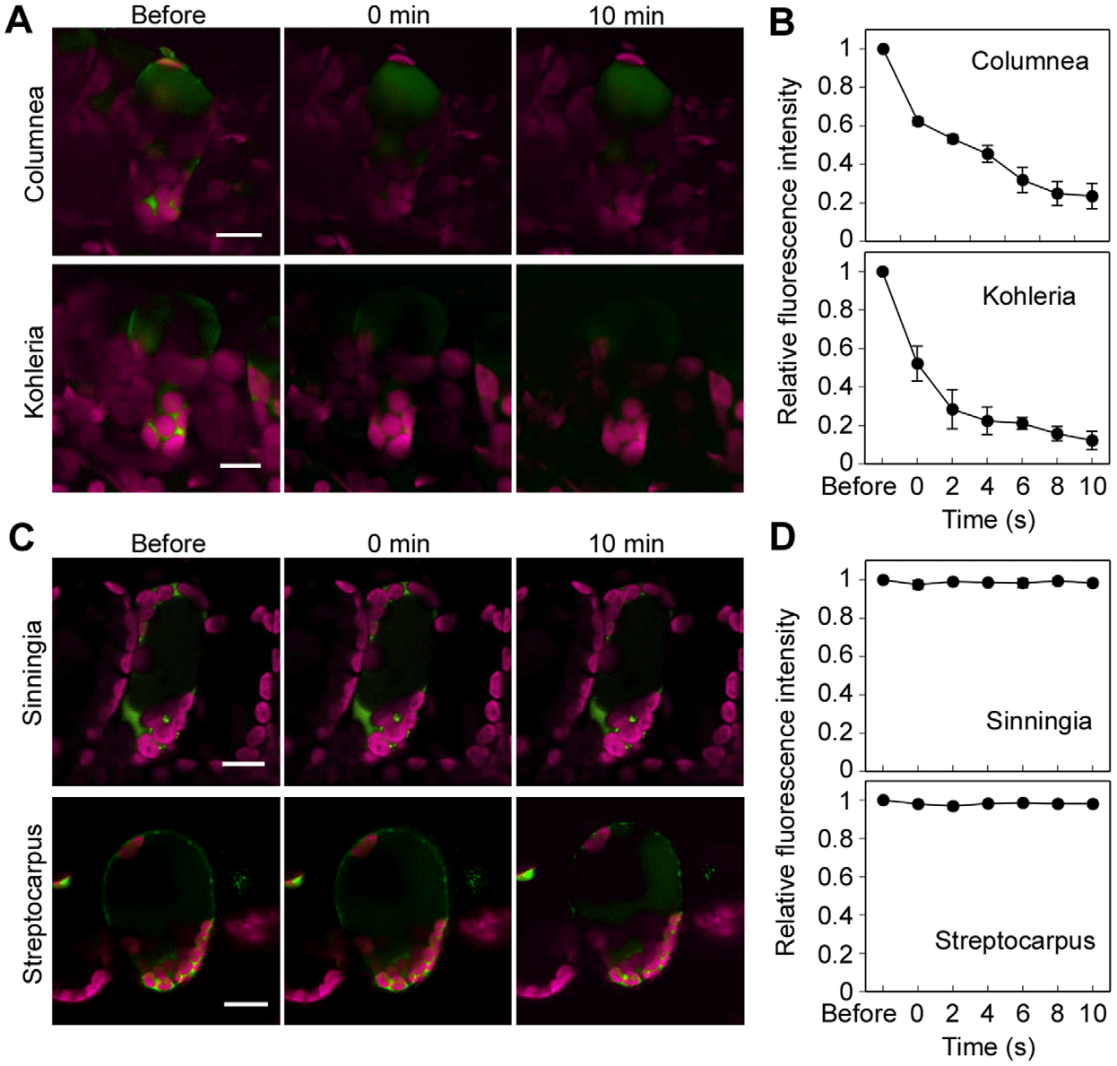
Cytosolic pH changes in palisade mesophyll cells of Gesneriaceae plants. Cytosolic pH changes in palisade mesophyll cell of sensitive species (*Columnea* sp. and *Kohleria warszewiczii*) and insensitive species (*Sinningia speciosa* and *Streptocarpus* sp.) induced by a rapid temperature decrease. (A) Palisade mesophyll cells of *Columnea* sp. and *Kohleria warszewiczii* stained with pH-sensitive fluorescent dye, BCECF-AM. (B) Relative changes of BCECF-AM fluorescence intensity in palisade mesophyll cell of *C.* sp. and *K. warszewiczii*. (C) Palisade mesophyll cells of *Sinningia speciosa* and *Streptocarpus* sp. stained with BCECF-AM. (D) Relative changes of BCECF-AM fluorescence intensity in palisade mesophyll cell of *S. speciosa* and *S.* sp. Images in (A) and (C) were taken before, immediately after (0 min), and 10 min after a temperature decrease from 30°C to 10°C and kept at 10°C for 1 min. Scale bar = 40 µm. The points and associated bars in (B) and (D) indicate mean intensity and standard error (n = 3).

Changes in cytoplasmic pH were also observed in the palisade mesophyll cells of Gesneriaceae species that are sensitive or insensitive to a rapid temperature decrease. Prior to the rapid temperature decrease from 30°C to 10°C, BCECF fluorescence was observed in the cytosol of palisade mesophyll cells (Fig. 8A, C). The fluorescence declined 10 min after the rapid temperature decrease in palisade mesophyll cells of sensitive *Columnea* sp. and *Kohleria warszewiczii* leaves (Fig. 8A); 10 min after the temperature decrease, the fluorescence intensity decreased by 76% and 70% in *C*. sp. and *K*. *warszewiczii,* respectively (Fig. 8B). A fluorescence decrease of BCECF was not observed when the temperature was maintained at 30°C (Fig. S2). Conversely, in the insensitive leaves of *Sinningia speciosa* and *Streptocarpus* sp., BCECF fluorescence was strongly emitted even after the temperature decrease (Fig. 8C) and its intensity was constant (Fig. 8D). Injury to the palisade mesophyll cells of sensitive Gesneriaceae also correlated with intracellular pH changes induced by a rapid temperature decrease from 30°C to 10°C.

## Discussion

We showed here that the leaves of saintpaulia were injured by a rapid temperature decrease (Fig. 1A). In previous studies, this leaf injury in saintpaulia had been observed in various strains; e.g. the original species *S*. *ionantha* [28], [45] and the cultivar ‘Ritali’ [30]. *Saintpaulia* cultivar ‘Iceberg’, which was used as our main experimental material, was also injured by temperature treatments that have been shown to affect other species. The severity of the injury was especially elevated (above 60%) when the temperature difference was greater than 15°C (Fig. 1B). Our results are consistent with a previous report [28]. Furthermore, the symptoms of the injury, i.e. leaf color change (Fig. 1A), palisade mesophyll cell shrinkage (Fig. 7C) and decline of chlorophyll fluorescence in chloroplasts (Fig. 2), were also in agreement with previous studies [30]–[32]. Thus, the leaf injury of the ‘Iceberg’ cultivar is similar to that found in the other saintpaulia strains.

Several studies have shown that the temperature conditions resulting in damage to saintpaulia leaf are different from other temperature injuries; this type of injury does not occur even at low temperature when the temperature is reduced slowly [28], [29]. For chilling sensitive plants, 5°C is a stressful temperature [1]. However, saintpaulia leaf was not injured even at 5°C when temperature decreased moderately [29]. *Saintpaulia shumensis*, one of the original species identified, inhabits a mountainous region of Tanzania where the mean minimum temperature of the coldest month is about 3°C with extreme minima below 0°C [22], [23], [45]. If this species was chilling sensitive, it would be impossible for it to survive in this habitat. Injury was not induced when the ambient air temperature decreased rapidly, because it took more than 15 min to lower the leaf temperature from 30°C to 15°C [28], although typical chilling injury is induced by moderate changes in leaf temperature [1]. These observations suggest that the injury in saintpaulia leaves is different to typical chilling injury.

We found that the severity of injury depended on the duration of the incubation period. The data presented in Figure 1C indicate that the sensitivity to rapid temperature decrease was enhanced by incubation at 30°C for over 10 min. These results suggested that the physiological conditions of the leaf are drastically changed within 10 min of the incubation; saintpaulia leaves might rapidly respond to ambient temperatures. Even in *Arabidopsis thaliana*, thought to have rapid acclimation mechanisms, it took 24 h to increase freezing tolerance [46]. In saintpaulia, leaves kept at 30°C for over 10 min might be more sensitive to 10°C than those kept for 5 min (Fig. 1C). Taking into consideration a previous report showing rapid hardening of saintpaulia leaves [29], we speculate that saintpaulia leaves have unique mechanisms to respond to ambient temperatures and reduce the extent of injury when temperature decreases slowly.

Chlorophyll fluorescence has been used as an indicator of environmental stress in plants, such as heat and cold stress, and herbicides [47]–[50]. A decline in chlorophyll fluorescence after leaf injury in saintpaulia has been reported [27], [31]. In this study, we continuously monitored chlorophyll fluorescence during the injury process and showed that decreases of øII and increases of NPQ occurred concurrently in saintpaulia leaves (Fig. 2). This irreversible decline of chlorophyll fluorescence was detected within a few seconds of the temperature decrease, although leaf discoloration could not be observed until a few minutes later. Chlorophyll fluorescence is also useful as an indicator to detect cell injury induced by rapid temperature decreases.

Under environmental stresses that limit fixation of CO_2_, such as heat, cold and salt, an excess of light energy suppresses the repair of photodamage to PSII [51], and these stresses might accelerate photoinhibition by inhibiting this repair [52]–[54]. Photoinhibition under stress can be detected using the chlorophyll fluorescence technique [55]. At unfavorable temperatures, a decrease in øII and increase in NPQ was observed in rice, barley, and a dipterocarp tree [56], [57]. In this study, we also observed low øII and high NPQ values in saintpaulia leaves after a rapid temperature decease (Fig. 2). This might be the result of photoinhibition and, if so, saintpaulia plants lack mechanisms to prevent photodamage when the temperature decreases rapidly. Interestingly, however, chloroplasts isolated from saintpaulia palisade mesophyll cells were not affected by a temperature decrease (Fig. 3). Chlorophyll fluorescence did not decline, and øII decreased reversibly even when the temperature was rapidly decreased, suggesting that the chloroplasts themselves may not be directly damaged by the rapid temperature decrease. These results indicated that, in palisade mesophyll cells, other cellular changes affecting chloroplasts occurred after the rapid temperature decrease.

It is known that the various cytosolic factors such as pH, Ca^2+^, and reactive oxygen species (ROS) can have an impact on photosynthesis [58]–[60]. Among these factors, we showed that cytosolic pH changed after a rapid decrease in temperature. Intracellular pH changes can be caused by various environmental stresses, such as low temperature, salt, high CO_2_, and hypoxia [61]–[65]. Chilling-induced cytoplasmic acidification of mung bean seedlings was caused by failure of pyrophosphate (PPi)-dependent H^+^ accumulation [66]. In this case, cytoplasmic and vacuolar pH monitored with a pH-sensitive fluorescent dye changed from 7.5 to 6.6 and from 5.1 to 5.7 after 8 h of exposure to 0°C, respectively [62]. Here, we observed that in palisade mesophyll cells of leaves sensitive to rapid temperature decrease, the cytosolic pH changed within 10 min of the temperature change (Figs. 4, 8). We could not determine changes in the absolute pH value, because palisade mesophyll cells have very thin cytosol and it is not easy to determine an exact pH value using fluorescence dyes. However, an abrupt decrease of fluorescence intensity implies rapid changes of intracellular pH. We predict that in the palisade mesophyll cells of sensitive species, acidic vacuolar contents leak into the cytosol, resulting in large changes of intracellular pH. Cytosolic pH changes after a rapid temperature decrease were observed only in palisade mesophyll cells (Fig. 4). This result indicated that sensitivity to rapid temperature decrease varied between different leaf tissues. However, at present we do not have any evidence to explain these differences in sensitivity. Cytosolic acidification disrupts various functions of organelles. For example, cytosolic acidification under high CO_2_ inhibits photosynthesis and causes a reduction in chlorophyll fluorescence [67]. In isolated spinach chloroplasts, O_2_ evolution reached a maximum around pH 7.6, but decreased when chloroplasts were suspended in media of higher or lower pH [64]. We showed that in chloroplasts isolated from saintpaulia leaves, fluorescence declined at weakly acidic pH (Fig. 5). Hence, chloroplasts in intact palisade mesophyll cells of saintpaulia may show a similar reduction in fluorescence upon acidification. From chloroplasts in spongy mesophyll cells, where intracellular pH did not change (Fig. 4), chlorophyll fluorescence was still observed even after a rapid temperature decrease [32].

The collapse of the vacuolar membrane was observed 10 min after the temperature decrease in palisade mesophyll cells (Fig. 6A, B). Vacuoles and vacuolar contents play a key role in programmed cell death (PCD) in plants [68]. Vacuolar processing enzyme (VPE), a protease that degrades vacuolar membranes, is involved in virus-induced hypersensitive cell death in tobacco plants [69]. After the collapse of the vacuolar membrane, vacuolar contents are released into the cytosol, resulting in rapid cell death. Our results strongly suggest that in palisade mesophyll cells of saintpaulia, the collapse of the vacuolar membrane is the event preceding chloroplast injury because cytosolic pH changed rapidly (Fig. 4). There is a report of electron microscopic observations in saintpaulia leaf cells after a rapid temperature decrease [30] in which the authors suggested that some invagination into the vacuole of tonoplast filled with cytosol could be seen. We suppose at present that these structures seem to suggest collapse of the vacuolar membrane, and this is supported by direct observations of the vacuolar membrane with fluorescent dye (Fig. 6) in the present study.

At present we do not know the identity of the signal for the collapse of the vacuolar membrane. One possibility is the rapid temperature change itself. It is well known that the lipid components of the bio-membrane change depending on the temperature, and therefore membrane fluidity may play a role in temperature sensing. Alternatively, temperature effects on the chloroplasts may lead to damage to the vacuole. ROS, which are thought to signal molecules that trigger PCD, are produced in chloroplasts when plants are exposed to temperature changes [70], and these may trigger collapse of vacuolar membrane resulting in intracellular pH changes. The involvement of ROS in saintpaulia leaf damage has been strongly implicated [33], [34] but it is not known how ROS damage the vacuolar membrane. The nature of the initial event is unclear because both changes in chlorophyll fluorescence and pH occur rapidly following a rapid temperature change. Here, we showed that the vacuoles collapsed during the initial phase of injury.

A more difficult question is “What is the role of such a physiological response to rapid temperature changes?” To consider this, we investigated the responses of some other Gesneriaceae species. Gesneriaceae is a large family comprising ca. 2000 species in ca. 150 genera distributed mostly in the tropics with a few species in temperate Europe and Asia [71]. The leaves of some species or cultivars of Gesneriaceae and Acanthaceae were also injured by the same temperature conditions causing discoloration of saintpaulia leaves [26]. However, the physiological mechanisms of those plants were not compared. In this study, we examined 29 species in 19 genera, and showed 10 species to be sensitive to rapid decreases in temperature resulting in injuries (Fig. 7A). According to phylogenetic analysis ([72], Fig. S3), genera with sensitive species were not necessarily genetically similar to each other. Indeed, both sensitive and insensitive species were found within the same genus in *Chirita* and *Streptocarpus*. These data suggest that differences in sensitivity to rapid temperature decrease may have evolved with climatic conditions in their individual habitats. The correlation between differences in sensitivity and environmental temperatures cannot be resolved without more microclimate data from each habitat.

The leaves of sensitive plants exhibited variations in shape, size, and thickness (Fig. 7A, C). The thick leaves of *Chirita tamiana*, *Columnea linearis*, *Columnea* sp., *Nematanthus maculatus*, *Gesneria pedicellaris*, and *Streptocarpus saxorum* had single or multiple layers of hypodermis beneath the upper epidermis (Fig. 7C). The hypodermis may act as a barrier against oscillation of heat when the leaf is exposed to sunflecks [73] or a water-storage tissue for adaptation to a dry climate [74]. However, leaves with a hypodermis were also injured by a rapid temperature decrease. Conversely, the leaves of *Seemannia sylvatica* and *Streptocarpus* sp., which lack a hypodermis, exhibited insensitivity and were not injured. Hence, sensitivity to rapid temperature decrease may not be determined by anatomical variations in leaves. Considering that only palisade mesophyll cells were injured in discolored leaves, sensitivity may be determined by the susceptibility of palisade mesophyll cells to rapid temperature decrease.

In conclusion, our results strongly suggest that the vacuoles of palisade mesophyll cells collapse during the initial phase of leaf injury. Stabilization of the vacuolar membrane may be essential to tolerate rapid decreases in temperature. The molecular basis of the injury and tolerance mechanisms are under investigation. Whether such temperature sensitivity has an important physiological role, or is only a genetic variation left during evolution, remains to be resolved.

## Supporting Information

Figure S1 **Cytoplasmic pH in palisade mesophyll cells of saintpaulia at constant temperature.** (A) Palisade mesophyll cells of saintpaulia leaf stained with BCECF-AM. Images of BCECF-AM (green) and chlorophyll fluorescence (magenta) were captured 0, 2 and 12 min after the start of observation at 25°C, respectively. (B) Relative changes of BCECF-AM fluorescence intensity at 25°C. Scale bar = 40 µm. The points and associated bars indicate mean intensity and standard error (n = 3).
(TIF)

Figure S2 **Cytoplasmic pH in palisade mesophyll cells of Gesneriaceae plants at constant temperature.** (A) Palisade mesophyll cells of *Columnea* sp. and *Kohleria warszewiczii* leaves stained with a pH-sensitive fluorescent dye, BCECF-AM. Images of BCECF-AM (green) and chlorophyll fluorescence (magenta) were captured 0, 2 and 12 min after of the start of observation at 30°C. Scale bar = 40 µm. (B) Relative changes of fluorescence intensity of BCECF-AM at 30°C. The points and associated bars indicate mean intensity and standard error (n = 3).
(TIF)

Figure S3 **Phylogenies of a subset of Gesneriaceae plants.** Phylogenetic trees based on ribosomal DNA internal transcribed spacer 1 (ITS1) sequences were constructed using the neighbor-joining (A) and maximum likelihood (B) methods. Numbers above branches are bootstrap percentages (1000 replicates). Bold branches indicate lineages sensitive to rapid temperature decrease. *Crossandra infundibuliformis* and *Hypoestes phyllostachya*, which belong to the Acanthaceae, were used as an outgroup.
(TIF)
