# Supplementary figures and images for: Sudden Collapse of Vacuoles in Saintpaulia sp. Palisade Cells Induced by a Rapid Temperature Decrease
Source: PLoS One. 2013 Feb 25;8(2):e57259. doi: 10.1371/journal.pone.0057259 (PMC3581458; doi:10.1371/journal.pone.0057259)

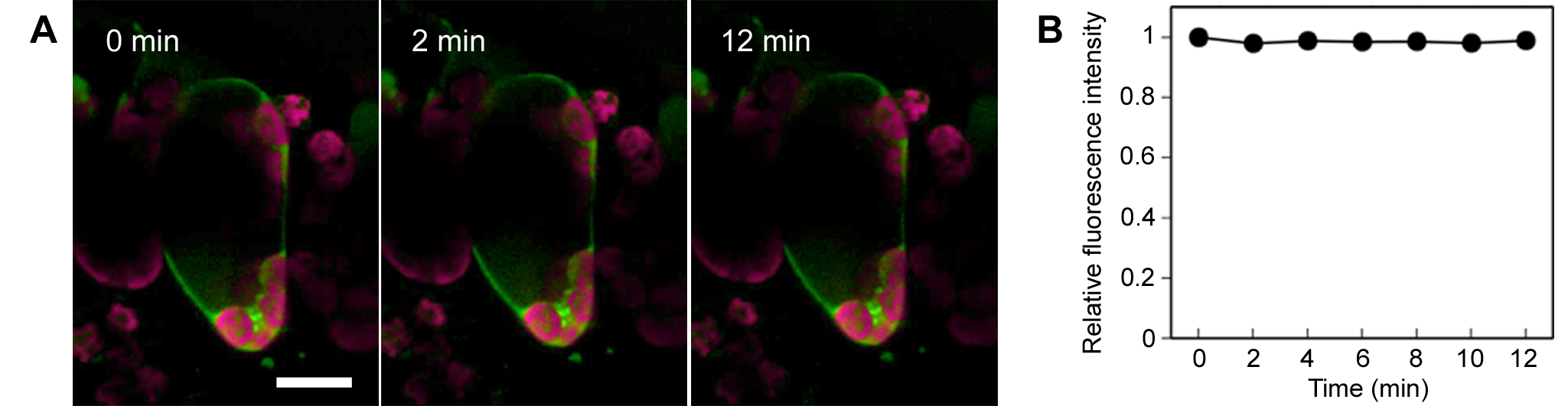

Supplement: Figure S1 — Cytoplasmic pH in palisade mesophyll cells of saintpaulia at constant temperature. (A) Palisade mesophyll cells of saintpaulia leaf stained with BCECF-AM. Images of BCECF-AM (green) and chlorophyll fluorescence (magenta) were captured 0, 2 and 12 min after the start of observation at 25°C, respectively. (B) Relative changes of BCECF-AM fluorescence intensity at 25°C. Scale bar = 40 µm. The points and associated bars indicate mean intensity and standard error (n = 3). (TIF) [file pone.0057259.s001.tif]

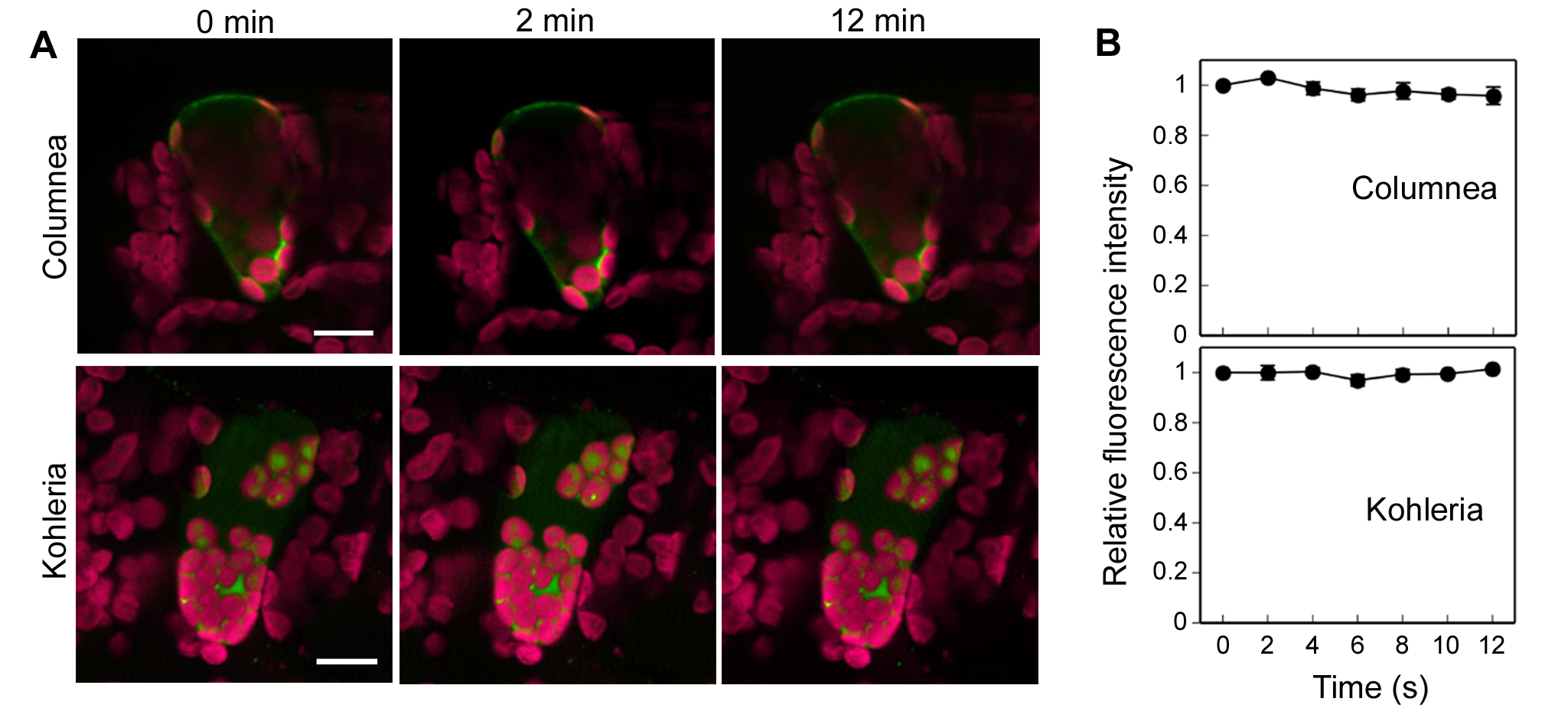

Supplement: Figure S2 — Cytoplasmic pH in palisade mesophyll cells of Gesneriaceae plants at constant temperature. (A) Palisade mesophyll cells of Columnea sp. and Kohleria warszewiczii leaves stained with a pH-sensitive fluorescent dye, BCECF-AM. Images of BCECF-AM (green) and chlorophyll fluorescence (magenta) were captured 0, 2 and 12 min after of the start of observation at 30°C. Scale bar = 40 µm. (B) Relative changes of fluorescence intensity of BCECF-AM at 30°C. The points and associated bars indicate mean intensity and standard error (n = 3). (TIF) [file pone.0057259.s002.tif]

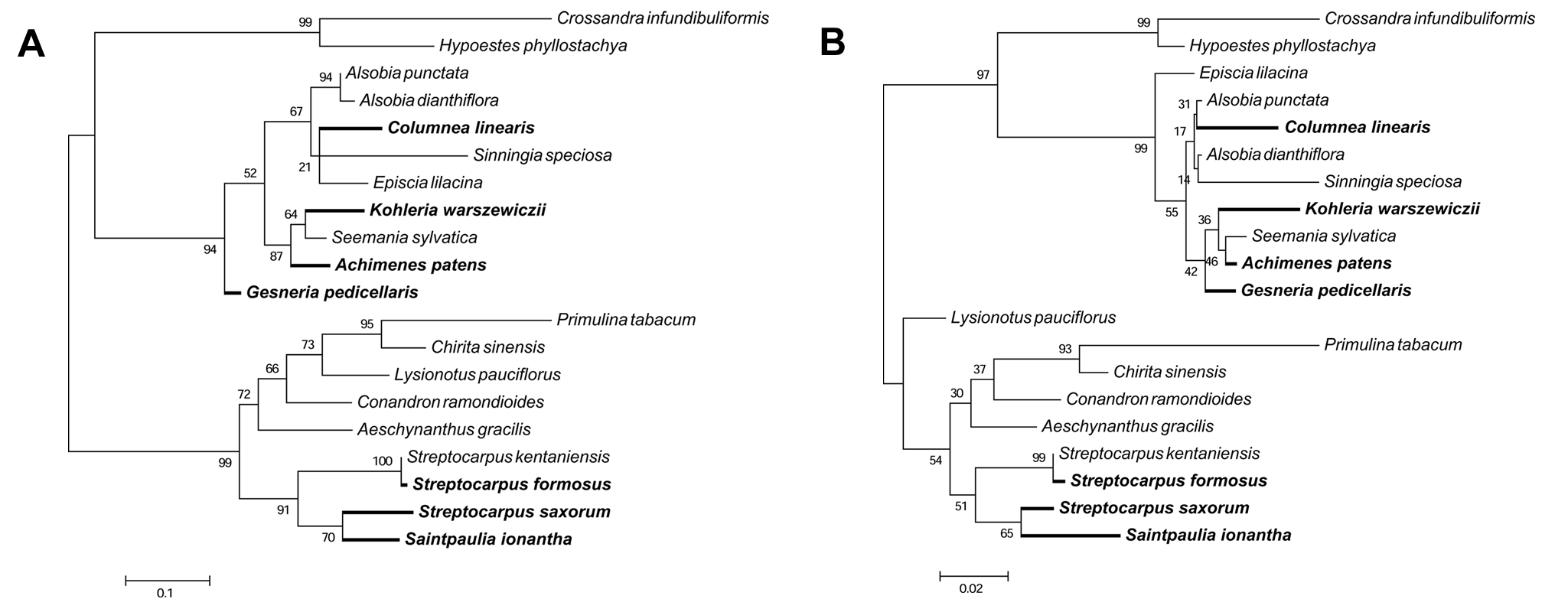

Supplement: Figure S3 — Phylogenies of a subset of Gesneriaceae plants. Phylogenetic trees based on ribosomal DNA internal transcribed spacer 1 (ITS1) sequences were constructed using the neighbor-joining (A) and maximum likelihood (B) methods. Numbers above branches are bootstrap percentages (1000 replicates). Bold branches indicate lineages sensitive to rapid temperature decrease. Crossandra infundibuliformis and Hypoestes phyllostachya, which belong to the Acanthaceae, were used as an outgroup. (TIF) [file pone.0057259.s003.tif]
